# Sonic hedgehog functions upstream of *disrupted-in-schizophrenia 1* (*disc1*): implications for mental illness

**DOI:** 10.1242/bio.012005

**Published:** 2015-09-24

**Authors:** Penelope J. Boyd, Vincent T. Cunliffe, Sudipto Roy, Jonathan D. Wood

**Affiliations:** 1 Sheffield Institute for Translational Neuroscience, Department of Neuroscience, University of Sheffield, 385a Glossop Road, Sheffield S10 2HQ, UK; 2 Institute of Molecular and Cell Biology, 61 Biopolis Drive, 138673, Singapore; 3 Bateson Centre, Department of Biomedical Science, University of Sheffield, Firth Court, Western Bank, Sheffield S10 2TN, UK; 4 Department of Biological Sciences, National University of Singapore, 14 Science Drive 4, 117543, Singapore; 5 Department of Pediatrics, Yong Loo Lin School of Medicine, National University of Singapore, 1E Kent Ridge Road, 119288, Singapore

**Keywords:** DISC1, Sonic Hedgehog, CNS, Mental illness

## Abstract

*DISRUPTED-IN-SCHIZOPHRENIA* (*DISC1*) has been one of the most intensively studied genetic risk factors for mental illness since it was discovered through positional mapping of a translocation breakpoint in a large Scottish family where a balanced chromosomal translocation was found to segregate with schizophrenia and affective disorders. While the evidence for it being central to disease pathogenesis in the original Scottish family is compelling, recent genome-wide association studies have not found evidence for common variants at the *DISC1* locus being associated with schizophrenia in the wider population. It may therefore be the case that DISC1 provides an indication of biological pathways that are central to mental health issues and functional studies have shown that it functions in multiple signalling pathways. However, there is little information regarding factors that function upstream of DISC1 to regulate its expression and function. We herein demonstrate that Sonic hedgehog (Shh) signalling promotes expression of *disc1* in the zebrafish brain. Expression of *disc1* is lost in *smoothened* mutants that have a complete loss of Shh signal transduction, and elevated in *patched* mutants which have constitutive activation of Shh signalling. We previously demonstrated that *disc1* knockdown has a dramatic effect on the specification of oligodendrocyte precursor cells (OPC) in the hindbrain and Shh signalling is known to be essential for the specification of these cells. We show that *disc1* is prominently expressed in *olig2*-positive midline progenitor cells that are absent in *smo* mutants, while cyclopamine treatment blocks *disc1* expression in these cells and mimics the effect of *disc1* knock down on OPC specification. Various features of a number of psychiatric conditions could potentially arise through aberrant Hedgehog signalling. We therefore suggest that altered Shh signalling may be an important neurodevelopmental factor in the pathobiology of mental illness.

## INTRODUCTION

Since its discovery in 2000, *DISRUPTED-IN-SCHIZOPHRENIA* (*DISC1*) has been one of the most widely studied genetic risk factors for mental illness. It was discovered through positional mapping of a translocation breakpoint in a large Scottish family where a balanced t(1;11) (q42.1;q14.3) translocation was found to segregate with schizophrenia and affective disorders (Millar et al., 2000). The *DISC1* gene contains 13 exons and encodes an 854 amino acid protein with a highly conserved C-terminal region containing multiple coiled-coil motifs. The translocation breakpoint is situated between exons 8 and 9, thereby disrupting the conserved coiled-coil region which is important for interaction with a number of proteins important in neurodevelopment, including NDEL1, NDE1 and LIS1 (Millar et al., 2003; Morris et al., 2003; Ozeki et al., 2003). The relevance of DISC1 to mental illness in the wider population remains a matter for fierce debate (Porteous et al., 2014; Sullivan, 2013), but functional studies have demonstrated that DISC1 has important roles in embryonic and adult neurogenesis (Chandran et al., 2014; Duan et al., 2007; Mao et al., 2009; Namba and Kaibuchi, 2010). Moreover, mice engineered to express *C*-terminally truncated human DISC1 (Hikida et al., 2007; Li et al., 2007; Pletnikov et al., 2008; Shen et al., 2008), containing a targeted deletion of exons 2 and 3 (Kuroda et al., 2011), harbouring a naturally occurring 25 base pair deletion in the *Disc1* gene (Gomez-Sintes et al., 2014; Juan et al., 2014) or carrying ENU-induced point mutations in *Disc1* (Clapcote et al., 2007) all show behavioural abnormalities. Moreover, interactions between DISC1 and environmental factors have been shown to modulate behaviour (Abazyan et al., 2010; Haque et al., 2012; Ibi et al., 2010; Niwa et al., 2013). Therefore, the study of DISC1 biology may provide fundamental insight into the complex interplay between genetic, developmental and environmental factors that underlie mental illness. It may be the case that while perturbation of DISC1 function is causal in the Scottish family, alterations in upstream and downstream signalling pathways may be more relevant to mental health issues in the wider population.

While considerable effort has gone into delineating the signalling pathways that are regulated by the DISC1 protein (Duan et al., 2007; Kim et al., 2009, 2012; Mao et al., 2009; Millar et al., 2005; Namba et al., 2011; Zhou et al., 2013) little is known regarding the factors that lie upstream of *DISC1*. One study, where analysis of the *DISC1* promoter was undertaken, revealed that it is a target of the forkhead transcription factor FOXP2 (Walker et al., 2012). In order to understand the neurodevelopmental functions of DISC1, a number of groups have investigated the developmental expression and functions of *disc1* in the zebrafish embryo (De Rienzo et al., 2011; Drerup et al., 2009; Wood et al., 2009). The studies from the Sive and Morris laboratories showed roles for *disc1* in embryonic neurogenesis and neural crest migration/differentiation respectively, while our studies revealed a novel requirement for *disc1* in the specification of oligodendrocyte precursor cells in the hindbrain (Wood et al., 2009). Subsequent studies have supported an important role for DISC1 in oligodendrocyte development in higher vertebrates (Hattori et al., 2014; Katsel et al., 2011; Seshadri et al., 2010; Shimizu et al., 2014), while interrogation of a CNS cell types exon array (Cahoy et al., 2008) by us revealed that among the CNS cell types analysed, *Disc1* was most highly expressed in OPCs. DISC1 has also been implicated in agenesis of the corpus callosum in humans (Osbun et al., 2011). Importantly, oligodendrocyte and white matter abnormalities are a widely reported feature of schizophrenia and affective disorders (Bernstein et al., 2015; Mahon et al., 2010; Takahashi et al., 2011; Tham et al., 2011), implying that white matter dysfunction may contribute to the complex aetiology of a range of mental disorders.

At around 2 days post-fertilisation (dpf), *disc1* is prominently expressed in a number of ventral regions in the zebrafish embryo, including the midline of the hindbrain, otic vesicle and developing cartilages of the branchial arches (Wood et al., 2009). Sonic hedgehog (Shh) is an important morphogen with essential roles in dorsal-ventral patterning during embryogenesis. Ablation of Shh leads to a failure to produce oligodendrocytes in the chick spinal cord (Orentas et al., 1999) and zebrafish hindbrain (Cunliffe and Casaccia-Bonnefil, 2006). Similarly, Shh has essential roles in the development of the otic vesicle and branchial arches in zebrafish (Hammond et al., 2003; Wada et al., 2005). Given that *disc1* appears to be predominantly expressed in a number of Shh-responsive tissues, we therefore reasoned that *disc1* may function downstream of Shh signalling. To test this hypothesis we have investigated the effects of genetic and chemical modulation of Shh signalling on *disc1* expression in the zebrafish embryo and used double-labelling methods to characterise *disc1*-expressing cells in the hindbrain. These studies suggest a critical role for Shh signalling in the development of *disc1*-expressing cells in the zebrafish CNS and other tissues.

## RESULTS

### Expression of *disc1* is altered in Shh pathway mutant embryos

**Fig. 1.**
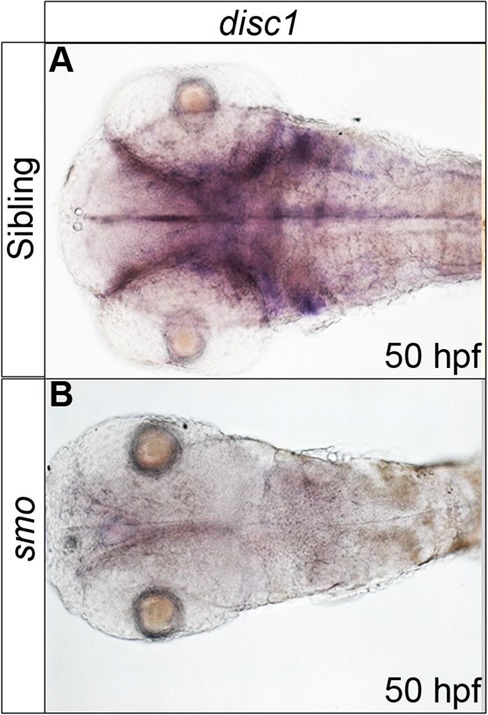
***disc1* expression is absent in *smo^b641^* mutant embryos at 50 hpf.** Whole mount *in situ* hybridization analysis for *disc1* expression in *smo^b641^* (B) revealed a near total loss of expression throughout the CNS and other tissues compared with sibling controls (A). Dorsal views, anterior to left.

To determine whether levels of Shh signalling influence *disc1* expression in the CNS, *disc1* expression was analysed in Hedgehog (Hh) pathway mutant embryos. We utilised three stable genetic mutant lines: *smo^b641^* loss-of-function mutants carrying a mutation in the Smoothened (Smo) receptor leading to complete loss of Shh signal transduction (Varga et al., 2001); *igu^ts294e^* mutants that have a mutation in DAZ-interacting protein 1 (DZIP1) leading to ciliary defects and subsequent aberrations in Shh signal transduction (Brand et al., 1996); and *ptch1;ptch2* loss-of-function double mutants which harbour mutations in both Patched1 and Patched2 leading to constitutive activation of Shh signalling (Koudijs et al., 2008). In the brain, prominent expression of *disc1* is observed in the ventral diencephalon, midline of the hindbrain, and dorsolateral regions of the hindbrain where the expression pattern resembles that of the radial glia/neural progenitor marker *fabp7a*/*blbp* (supplementary material Fig. S1). In both *smo^b641^* (Fig. 1) and *igu ^ts294e^* (supplementary material Fig. S2) mutants, *disc1* expression was dramatically reduced in all of these regions of the brain, as well as in non-CNS regions such as the tissue surrounding the otic vesicles (Fig. 1B; supplementary material Fig. S2B), compared with control sibling embryos (Fig. 1A; supplementary material Fig. S2A).

**Fig. 2.**
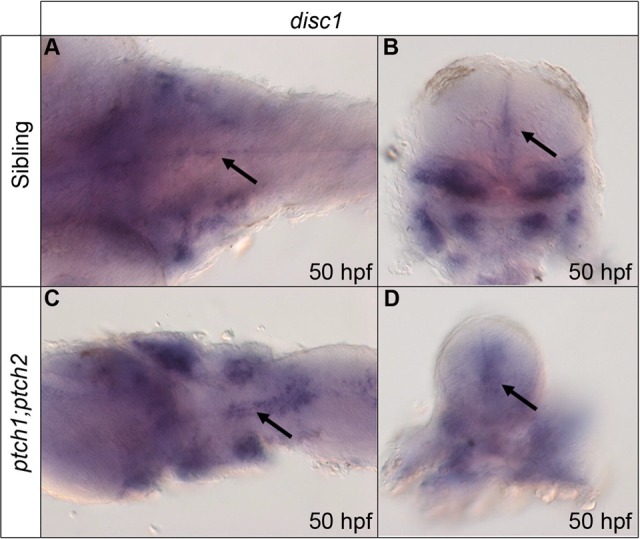
**Expression of *disc1* at 50 hpf is up-regulated around the midline of the hindbrain in response to increased Shh signalling.** Expression was analysed using whole mount *in situ* hybridization. Dorsal, anterior to left (A,C) and transverse sectional (B,D) views show increased *disc1* expression around the midline of the hindbrain in *ptch1;ptch2* mutants (C,D) compared with siblings (A,B). Arrows indicate hindbrain midline *disc1* expression in transverse sectional views.

In *ptch1;ptch2* double homozygous mutant embryos (Fig. 2C,D), *disc1* expression was strongly increased around both sides of the midline of the hindbrain compared to sibling embryos (Fig. 2A,B) at 50 hpf. In these *ptch1;ptch2* double mutants, the expression of *disc1* increased from a thin strip of expression in the midline seen in controls (Fig. 2A) to prominent strips of expression on either side of the midline in double mutants (Fig. 2C), with both the number of cells labelled and intensity of cell labelling appearing to be increased in the hindbrain. Analysis of transverse sections taken through the hindbrain also demonstrated increased *disc1* expression around the midline (compare Fig. 2D with B). Comparison of *disc1* expression in non-CNS tissues was complicated by the highly dysmorphic nature of the *ptch1;ptch2* double mutants, with the loss of organised staining in the developing jaw cartilages being particularly notable (Fig. 2B,D). To determine whether constitutive activation of Shh signalling influences OPC development, *in situ* hybridisation for *olig2* was performed on *ptch1;ptch2* double mutants. OPCs form a stereotypical pepperpot distribution pattern, which is highly consistent between wild type embryos (supplementary material Fig. S3A). However this stereotypical pattern was lost in the *ptch1;ptch2* double homozygous mutants (supplementary material Fig. S3C), and the distribution of OPCs differed between individual double mutant embryos. The normal initial restriction of OPCs to ventral regions of the hindbrain (supplementary material Fig. S3B) was lost, and *olig2*-expressing cells were observed in both ventral and dorsal regions of the hindbrain in a radial distribution (supplementary material Fig. S3D). Therefore, increased Shh signalling leads to increased *disc1* expression and an altered distribution of OPCs in the hindbrain.

**Fig. 3.**
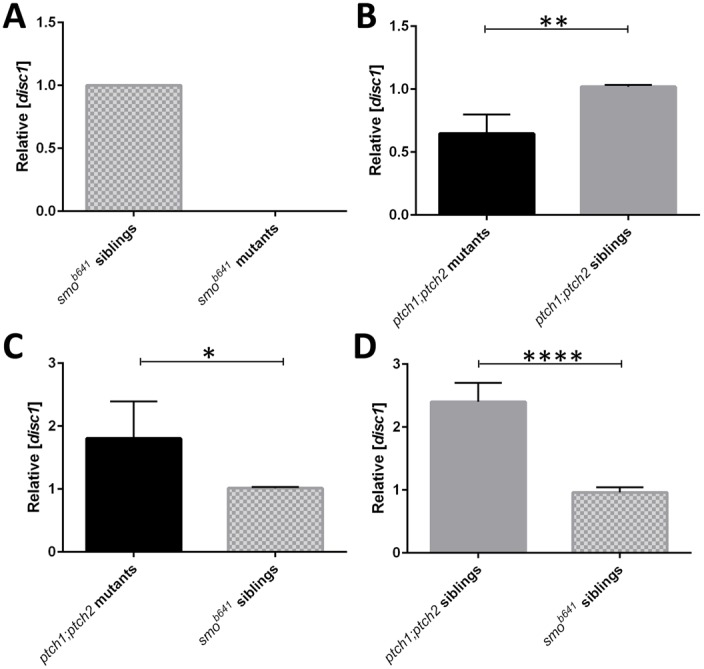
**Reverse-transcription quantitative PCR (RT-qPCR) analysis demonstrates that *disc1* expression is altered in sonic hedgehog pathway mutants.** (A) Graphical representation demonstrating that expression of *disc1* was below the threshold for detection in *smo^b641^* mutant embryos at 50 hpf. (B) Expression of *disc1* was 0.65-fold lower in *ptch1;ptch2* double mutants than siblings (*P*=0.0027). Both *ptch1;ptch2* mutant (C; 1.8-fold, *P*=0.035) and sibling (D; 2.4-fold, *P*<0.0001) pools show increased *disc1* expression relative to *smo^b641^* sibling embryos at 50 hpf. (*n*=4; *P<0.05, **P<0.005, ****P<0.0001; error bars show s.e.m.).

To confirm that expression of *disc1* is responsive to levels of Hh signalling, reverse-transcription quantitative PCR (RT-qPCR) was performed using total RNA prepared from pools of 20 *smo^b641^* and *ptch1;ptch2* mutants as well as pools of sibling controls. PrimeTime Mini qPCR assays were performed for *disc1* and *eukaryotic translation elongation factor 1 alpha 1b* (*eef1a1b*), the latter serving as a reference gene for normalisation (Flinn et al., 2013; Tang et al., 2007). While *disc1* expression was readily detected in pools of *smo^b641^* sibling embryos, *disc1* expression failed to reach the threshold level for detection in *smo^b641^* mutant embryos, confirming the loss of *disc1* expression in the absence of Shh signal transduction. This is represented graphically in Fig. 3A. Surprisingly, qPCR assays demonstrated that the relative level of *disc1* expression in pools of *ptch1;ptch2* mutants was slightly reduced compared with sibling controls (Fig. 3B). The developing jaw cartilages are the strongest site of *disc1* expression in wild type embryos, but these fail to form normally in *ptch1;ptch2* double mutants (compare Fig. 2B and D), potentially masking any increase in *disc1* expression in the double mutants compared with their siblings. In addition, these pools were sorted purely on the basis of the gross morphological differences apparent in the double homozygous mutants, since single homozygous mutants for both *ptch1* and *ptch2* were indistinguishable from wild type and heterozygous siblings at the required stage. Therefore sibling pools will have contained a mixture of genotypes for *ptch1* and *ptch2*, including individual homozygous mutants for both *ptch1* and *ptch2* in conjunction with heterozygosity for the other paralogue. Individual homozygous mutant *ptch1* and *ptch2* embryos show subtle morphological phenotypes such as eye and pigmentation defects around 3 dpf that are caused by increased levels of Hh signal transduction (Hammond and Schulte-Merker, 2009; Koudijs et al., 2008, 2005), and the additive effect of loss of wild type *ptch1* and *ptch2* alleles on expression of Shh target genes has been well documented; for example, see Fig. 3 in (Koudijs et al., 2008). Consequently, more than half of the embryos in the *ptch1;ptch2* sibling pool would be expected to have elevated levels of Hh signalling compared to wild type embryos, so we therefore compared *disc1* expression in the *ptch1;ptch2* sibling and mutant pools with the *smo^b641^* sibling pool. These comparisons showed that *disc1* expression was around 1.8- and 2.4-fold higher respectively in the *ptch1;ptch2* mutant and sibling pools compared with the *smo^b641^* sibling pool (Fig. 3C,D), confirming that *disc1* expression is responsive to increased levels of Shh signalling.

### *disc1* is specifically expressed in *olig2*-positive midline cells and is responsive to Shh signalling

**Fig. 4.**
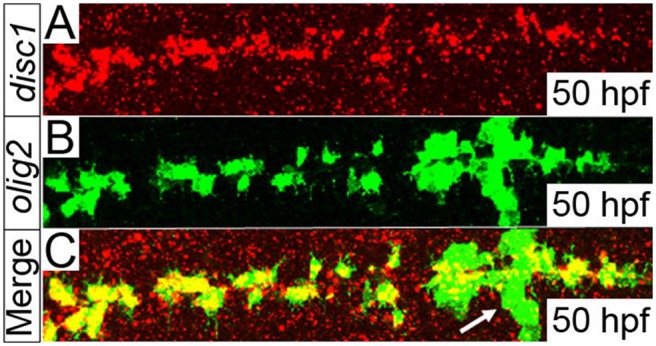
***disc1* is expressed in *olig2*-positive cells located around the midline of the hindbrain at 50 hpf.** A fluorescent *in situ* stain for *disc1* mRNA (A, red) was performed in tandem with immunofluorescence staining for EGFP (B, green) on fixed *Tg(olig2:egfp)* embryos. The merged image shows clear overlap of the red and green signals in cells distributed along both sides of the midline of the hindbrain, but not in the abducens motor neurons (arrow). Dorsal views, anterior to left.

We previously showed that knock down of *disc1* in the zebrafish embryo leads to oligodendrocyte precursor cell (OPC) specification defects in the hindbrain (Wood et al., 2009). We demonstrated that *disc1* was expressed in the same region as *olig2*-positive cells using *in situ* hybridisation, but co-expression of *disc1* in *olig2*-positive cells was not confirmed using double-staining methods. Therefore, a fluorescent *in situ* stain for *disc1* mRNA was performed in tandem with immunofluorescent staining for EGFP in the *Tg(olig2:egfp)* line to confirm this and to identify the *disc1*-expressing cells in the hindbrain. The expression of *disc1* was most prominent in *olig2*-positive cells along the midline of the hindbrain at 50 hpf (Fig. 4). A high background signal was consistently obtained when performing fluorescent *in situ* staining for *disc1* mRNA, but the signal was clearly intensified in the EGFP-positive cells around the midline. Furthermore, the complete loss of *disc1* expression around the midline of the hindbrain in *smo^b641^* and *igu^ts294e^* mutants (Fig. 1; supplementary material Fig. S2), where the specification of these *olig2*-positive cells is inhibited, is consistent with *disc1* being specifically expressed in these cells. In transverse section (Fig. 6), these cells show morphology consistent with that of *olig2*-positive neuroepithelial progenitors described elsewhere (Zannino and Appel, 2009).

**Fig. 5.**
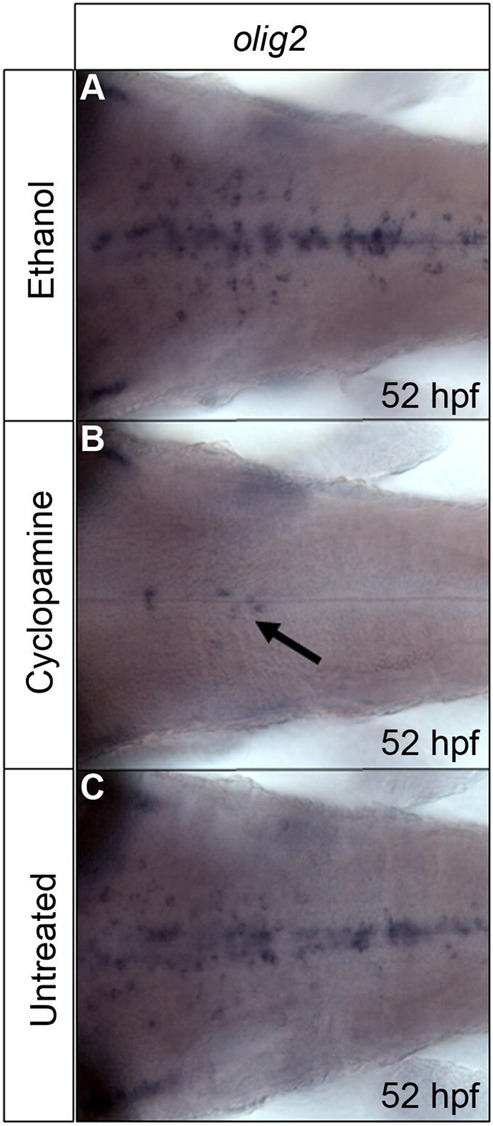
**Cyclopamine treatment leads to a dramatic reduction in the number of *olig2*-positive cells specified in the hindbrain.** Cyclopamine (50 µm) was applied to wild type embryos from 32 hpf then embryos analysed for *olig2* expression at 52 hpf. Cyclopamine-treated embryos (B) show a greatly reduced number of *olig2*-positive cells (arrow) compared with 0.5% ethanol treated (A) and untreated (C) control embryos. *n*=40, 20 embryos per group, data pooled from 2 independent experiments.

**Fig. 6.**
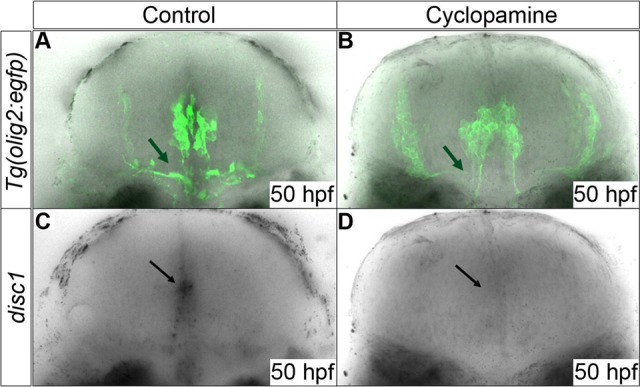
**Expression of *disc1* in EGFP expressing cells is down-regulated by cyclopamine treatment.** Cyclopamine (40 µm) was applied to *Tg(olig2:egfp)* embryos from 34 hpf prior to the main period of OPC proliferation and migration. Cyclopamine-treated embryos (D) showed a loss of midline *disc1* expression (black arrows) at 50 hpf compared to untreated embryos (C). Comparison of B with A shows a loss of EGFP-positive OPCs as a result of chemical inhibition of Shh signal transduction (green arrows). 10 animals were imaged from each group. All images are of transverse sections.

Given that Shh signalling is required for specification of this population of cells, we sought to demonstrate that inhibition of Shh signal transduction after their specification has been initiated, leads to loss of *disc1* expression. In order to test this, cyclopamine, a widely used chemical inhibitor of Shh signal transduction, was applied to wild type embryos from 32 hpf. This is after the initial specification of these progenitors in rhombomeres 5/6 has occurred, but prior to the main period of hindbrain OPC specification and migration (Zannino and Appel, 2009). Control embryos were either untreated or treated with solvent (0.5% ethanol) alone. Embryos were then fixed at 52 hpf and the specification of *olig2*-positive cells analysed by *in situ* hybridisation (Fig. 5). As expected, cyclopamine treatment led to a dramatic reduction, but not a complete ablation, in the number of *olig2*-positive cells in the hindbrain (Fig. 5B), compared with both ethanol-treated (Fig. 5A) and untreated (Fig. 5C) control embryos. To determine the effect of cyclopamine treatment on *disc1* expression, *Tg(olig2:egfp)* embryos were exposed to cyclopamine from 34 hpf, fixed at 50 hpf and then analysed for *disc1* mRNA expression using *in situ* hybridisation and EGFP expression using immunofluorescence. While EGFP expression was clearly observed in cells around the midline in both cyclopamine-treated and control embryos (Fig. 6A,B), there was complete loss of *disc1* expression in the EGFP-expressing cells in cyclopamine-treated embryos (Fig. 6D) when compared to control embryos (Fig. 6C). This suggests that *disc1* expression in these progenitors requires active Shh signal transduction. If Shh signals to drive *disc1* expression in these cells, then they need to have the ability to respond to this signalling pathway. Primary cilia play an essential role in transducing the Shh signal in many cell types. Analysis of primary cilia on EGFP^+^ cells in the *Tg(olig2:egfp)* line using an antibody against ADP-ribosylation factor-like protein 13B (Arl13b), which is a component of ciliary membranes and required for ciliogenesis (Zhang et al., 2013), clearly showed the presence of primary cilia on these cells at 36 hpf (supplementary material Fig. S4A). In addition to there being a loss of *disc1* expression around the midline in cyclopamine-treated embryos, a lack of EGFP-positive cells with a morphology resembling that of OPCs in the ventral hindbrain was apparent (green arrows in Fig. 6A and B). This is as might be expected, since the role of Shh signalling in OPC specification is well established.

### Cyclopamine treatment mimics the effect of *disc1* knockdown on OPC specification

**Fig. 7.**
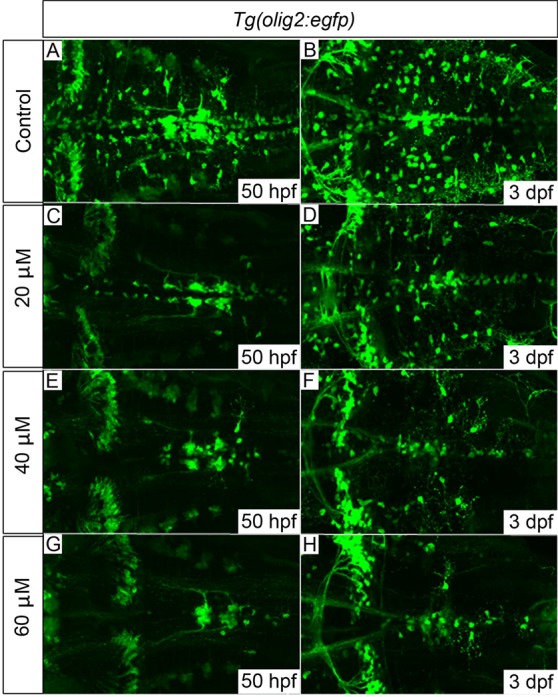
***Tg(olig2:egfp)* embryos treated with cyclopamine from 34 hpf show dose-dependent OPC proliferation and migration defects.** OPCs failed to migrate fully to uniformly populate the hindbrain in cyclopamine-treated embryos (C-H) compared with control embryos (A,B). The development of *olig2*-positive cerebellar neurons (clearly visible towards the left of panel H) and the abducens motor neurons (axonal projections clearly visible in G) appeared normal, even with the highest dose of 60 µM, which suggests that their development is largely Shh-independent. 20 embryos per treatment group; images show dorsal views, anterior to left. Quantification of OPC numbers is shown in supplementary material Fig. S5.

We previously demonstrated that *disc1* knockdown inhibits the specification of hindbrain OPCs after their initial specification in rhombomeres 5/6 (Wood et al., 2009). To confirm that inhibition of Shh signalling at the appropriate developmental stage gives a similar phenotype, cyclopamine (20–60 µM) was applied continuously to *Tg(olig2:egfp)* embryos (*n*=20 per group) from 34 hpf, and fixed specimens analysed at either 50 hpf (*n*=10) or 3 dpf (*n*=10). A clear dose-dependent reduction in the number of *olig2*-positive cells specified along the midline at 50 hpf was observed (Fig. 7C,E,G). In addition, cells with the morphology of OPCs failed to migrate laterally to uniformly populate the hindbrain at 3 dpf (Fig. 7D,F,H). Individual OPCs outside the initial zone of *olig2* promoter-driven EGFP expression in r5/6 were counted in a z-stack projection of the entire hindbrain. This quantification of OPC numbers (*n*=5 per treatment group) showed that the number of OPCs in this region reduced dramatically with increasing cyclopamine concentration (supplementary material Fig. S5). The overall morphology of the cyclopamine-treated embryos at both 50 hpf and 3 dpf was grossly normal, with few overt signs of toxicity such as CNS necrosis when cyclopamine was applied continuously at these doses. In addition, the development of GFP-positive abducens motor neuron axons (Fig. 7G) and cerebellar neurons (Fig. 7H) appeared normal, even at the highest dose. These results are consistent with the known role of Shh signalling in hindbrain OPC specification, and mimics the previously described effect of *disc1* knockdown on OPC development.

## DISCUSSION

We have provided evidence to demonstrate that Shh signalling functions upstream of *disc1* in oligodendrocyte development in the zebrafish hindbrain. Expression of *disc1* was absent in the hindbrain of *smo^b641^* mutants and increased in *ptch1;ptch2* double homozygous mutants; cyclopamine treatment abolished *disc1* expression in *olig2*-positive neuroepithelial progenitors in the midline of the hindbrain, and cyclopamine treatment over an appropriate timeframe mimicked the effect of *disc1* knockdown on OPC specification that we previously reported (Wood et al., 2009). While the loss of *disc1* expression in the midline of the hindbrain in *smo^b641^* mutants most likely represents a loss of *olig2*-positive neural progenitors, treatment with cyclopamine after the initial specification of this progenitor population had taken place was found to inhibit *disc1* expression in these cells. We therefore suggest that *disc1* expression in hindbrain neuroepithelial progenitors is driven by Shh in order to promote OPC specification. It remains to be determined whether the striking loss of *disc1* expression observed in other brain regions and in non-CNS tissues, such as the region surrounding the otic vesicles, reflects specification defects, transcriptional effects or a combination of both. However, given the apparent absence of *disc1* expression in *smo^b641^* mutants at 2 dpf, it appears that *disc1* may function downstream of Shh signalling in multiple cell types.

Canonical Hh signalling via its receptor Ptc1 and disinhibition of Smo culminates in transcriptional events mediated by the Gli family of transcription factors. One question that arises is whether *disc1* is a primary or secondary target of Gli family transcription factors? Bioinformatic analysis of the *disc1* promoter region did not find any high affinity Gli consensus-binding sites (GACCACCCA), but the ability of low affinity sites to efficiently activate transcription has been documented (Winklmayr et al., 2010). *Olig2* is a known target of Gli activators (Vokes et al., 2007; Wang et al., 2013), and a recent study found that *Disc1* is a direct target of Olig2 in spinal cord motor neuron progenitors and OPCs (Satoh et al., 2015), showing 89.8 and 23.5-fold enrichment respectively in ChIP-Seq datasets. We have found that morpholino-mediated knockdown of *olig2* abolishes *disc1* expression in the midline of the hindbrain (results not shown), but *olig2* function is known to be required for OPC development (Park et al., 2002), so this again may represent a block in the specification of the relevant progenitor population. One other known regulator of *DISC1* expression, FOXP2, has been suggested to interact with Shh signalling in the CNS (Carney et al., 2010; Chiu et al., 2014). Canonical Shh signalling emanates from primary cilia in vertebrates and the presence of these organelles was demonstrated on *olig2*-positive cells around the midline prior to the main phase of OPC specification. DISC1 has been shown to interact with PDE4B to regulate cAMP signalling (Millar et al., 2005), with GSK3β (Mao et al., 2009) and with KIAA1212 to regulate mTOR/AKT signalling (Kim et al., 2009, 2012). All of these signalling pathways are important mediators of Shh signal transduction, thereby implying that DISC1 may itself interface with Shh signalling. Furthermore, DISC1 has been suggested to associate with primary cilia (Marley and von Zastrow, 2010) and interacts with TRAF3IP1/MIPT3 (Morris et al., 2003), which has important roles in ciliogenesis (Li et al., 2008; Omori et al., 2008). These studies, linking DISC1 to primary cilia, further support the idea that DISC1 function may be linked to Shh signalling.

Given the wealth of data that has now accumulated regarding the neurodevelopmental functions of DISC1, the evidence for it being central to disease pathogenesis in the original Scottish family is compelling (Porteous et al., 2014). However, there is an apparent disconnect with genome-wide association studies, which have not linked common variation at the *DISC1* locus with schizophrenia or affective disorders in the wider population (Sullivan, 2013). It may therefore be the case that DISC1 provides insight into the developmental pathways and mechanisms that are central to mental health issues, and our findings lead us to propose that Shh signalling is an interesting candidate that warrants further investigation. A number of features of mental illnesses and the drugs used to treat them can be linked to Shh signalling. As discussed previously, oligodendrocyte and white matter abnormalities have been widely reported in schizophrenia and affective disorders (Bernstein et al., 2014; Mahon et al., 2010; Takahashi et al., 2011; Tham et al., 2011), and the importance of Shh signalling in oligodendrocyte development is well documented. Craniofacial dysmorphology has long been appreciated as a feature of schizophrenia, and has been taken as evidence to support the neurodevelopmental origins of this devastating condition (Lane et al., 1997). Many craniofacial features are derived from the highly migratory cranial neural crest during development, and important insights into conserved mechanisms of craniofacial development have been obtained using the zebrafish (Knight and Schilling, 2006). Hh signalling is one of the fundamental developmental pathways required for correct neural crest patterning (for review see Mishina and Snider, 2014) and *disc1* is prominently expressed in neural crest cells (Drerup et al., 2009; Wood et al., 2009), so the craniofacial abnormalities reported in schizophrenia may reflect altered Shh signalling, possibly via DISC1. Existing antipsychotic medications target dopamine receptors, and it has long been appreciated that Shh is essential for the development of midbrain dopaminergic neurons (Sillitoe and Vogel, 2008). Therefore, modified Shh signalling in the midbrain might be predicted to affect dopaminergic neuron development, potentially increasing susceptibility to psychiatric disease. Similarly, Shh signalling is essential for correct specification of hindbrain serotonergic neurons, which have been implicated in bipolar disorder and depression (Alenina et al., 2006). Modulation of Shh signalling has also been shown to be an effect of a number of antipsychotic drugs including clozapine, chlorpromazine and haloperidol (Lauth et al., 2010). Hence, some of the effects (or side effects) of these medications may ensue from altered Shh signalling .

Further evidence supporting involvement of Hh signalling in psychiatric disease has come from analysis of mouse models and human genetic studies. Male *Desert hedgehog* (*Dhh*) knockout mice show increased anxiety-like and depressive behaviour in tests such as the forced swim test (Umehara et al., 2006). More recently, the Hh pathway agonist SAG1.1 was shown to correct some of the structural and cognitive deficits seen in the Tg65Dn mouse model of Down syndrome (Das et al., 2013). A single injection of SAG1.1 on the day of birth gave a transient increase in the proliferation of cerebellar granule cells, and was able to correct the deficit in granule cell density seen in the Tg65Dn mouse. SAG1.1 treated mice also showed improved hippocampal long-term potentiation and correction of learning deficits in the Morris water maze compared with untreated mice, demonstrating improved hippocampal function in response to transient Hh pathway activation. Deletion and mutation of *PTCHD1*, which encodes a putative hedgehog ligand receptor, is associated with X-linked mental retardation and autism spectrum disorders (Chaudhry et al., 2015; Noor et al., 2010; Pinto et al., 2010). Finally, longitudinal study of Old Order Amish (OOA) families showing co-segregation of Ellis–van Creveld dwarfism (EvC) and bipolar affective disorder (BPAD) found that EvC confers protection against bipolar disorder (Ginns et al., 2014). Evc and its partner Evc2 are positive mediators of Shh signalling that form a complex with Smo to transduce the Hh signal via primary cilia (Blair et al., 2011; Dorn et al., 2012; Yang et al., 2012), suggesting that impaired Hh signalling in EvC patients may be protective against BPAD. Therefore, the pathways and mechanisms through which Shh signalling may act to regulate mood and behaviour are likely to be extremely complex. We have shown that *disc1* appears to function downstream of Shh in the zebrafish CNS, and it is of note that some of the signalling pathways that have been shown to be regulated by DISC1 (e.g. cAMP, GSK3 and Akt) are also known to be important in Shh signal transduction. Taken together, these studies provide evidence to support the notion that altered Shh signalling may be an important developmental factor in the pathobiology of mental illnesses and could be an alternative target for therapeutic drug development for these disorders, although establishment of appropriate time windows for intervention and the potential for oncogenic effects would merit caution.

## MATERIALS AND METHODS

### Zebrafish husbandry

The *Tg(olig2:egfp)* (Shin et al., 2003), *ptch1;ptch2* (Koudijs et al., 2008), *igu*(*dzip1)^ts294e^* (Brand et al., 1996) and *smo^b64^*^1^ (Varga et al., 2001) strains were raised and maintained at the Bateson Centre, University of Sheffield, UK and the Institute of Molecular and Cell Biology (IMCB) Singapore in AVA-approved aquaria. The *Tg(shha:gfp)* line (Ertzer et al., 2007) was maintained at the IMCB, Singapore. Adult zebrafish were maintained with a 14 h light/10 h dark cycle at 28°C according to standard protocols and were mated using spawning tanks and through pair mating in individual cross tanks (Nusslein-Volhard and Dahm, 2002). All procedures involving experimental animals were performed in compliance with local and national animal welfare laws, guidelines and policies.

### Immunostaining

Embryos were fixed using 4% paraformaldehyde and immunostaining performed using standard procedures (Schulte-Merker, 2003). Rabbit anti-GFP antibody (Torrey Pines Biolabs) was used at 1:1000 dilution, AlexaFluor488 donkey anti-rabbit secondary antibody (Abcam) was used at 1:500 dilution. When required, immunostaining was performed after RNA *in situ* hybridisation.

### Whole-mount *in situ* hybridisation

Digoxigenin-labelled antisense riboprobes were prepared using T7 and SP6 polymerases as described by the manufacturer (Roche Life Science). Templates used for generation of *olig2* (Cunliffe and Casaccia-Bonnefil, 2006) and *disc1* (Wood et al., 2009) probes are described elsewhere. Colormetric whole-mount *in situ* hybridisation was performed using standard procedures (Westerfield, 2000). Fluorescent *in situ* hybridisation staining was carried out using the Tyramide Signal Amplification (TSA™) system as described by the manufacturer (PerkinElmer).

### Cyclopamine treatment

Cyclopamine was obtained from Toronto Research Chemicals and added to embryos from a 10 mM stock prepared in ethanol. Dechorionated embryos (20 animals per group) were treated in E3 medium containing 20–60 µM cyclopamine from 32–34 hpf and kept in cyclopamine solution until the desired time point. Embryos were then fixed in 4% paraformaldehyde for staining.

### Reverse-transcription quantitative PCR (RT-qPCR)

Total RNA was isolated from pools of 20 embryos at 50 hpf using TRIZOL reagent (Life Technologies) and cDNA prepared using SuperScript III Reverse Transcriptase (Life Technologies). PrimeTime Mini qPCR 5′ nuclease assays for *disc1* and eukaryotic translation elongation factor 1 alpha 1b (*eef1a1b*), the latter serving as a reference gene for normalisation (Flinn et al., 2013; Tang et al., 2007), were designed using the Integrated DNA Technologies Real Time PCR tool (http://eu.idtdna.com/Scitools/Applications/RealTimePCR/). Assays were provided in single tubes, each containing a forward primer, a reverse primer and qPCR probe, optimised for use at a 1:20 dilution. Primer and probe sequences were as follows:

*disc1* forward primer, 5′-CGAGTGCTGAGTTTGTCCATC-3′;

*disc1* reverse primer, 5′-ATTTCCTGATACGGCTGTGAG-3′;

*disc1* probe, 5′6-FAM/CGTTTCGCT/ZEN/CTCGCTGACCTTCT/3′IABkFQ-3′;

*eef1a1b* forward primer, 5′-GGCTGGTGTTGGTGAATTTG-3′;

*eef1a1b* reverse primer, 5′-TTCTGGCTATAATTGGGCTCC-3′,

*eef1a1b* probe, 5′6-FAM/CGTGGGCGT/ZEN/TAACAAGATGGACTCTA/3′IABkFQ.

Each assay yielded a single product of the predicted size. For each reaction, 12.5 ng of cDNA was added to a 10 µl reaction containing 0.5 µl of 20× PrimeTime assay mix (Integrated DNA Technologies), 5 µl of Brilliant III Ultra-Fast QPCR Master Mix (Agilent Technologies) and 3.5 µl of nuclease-free water in a 96 well plate. All reactions were performed in triplicate. Quantitative PCR was performed using the Bio-Rad C1000 Touch thermal cycler with CFX96 Real-Time PCR detection system. All reactions were denatured at 95°C for 3 min followed by 40 cycles at 95°C for 10 s, 60°C for 30 s. Data was exported and analysed using the Bio-Rad CFX Manager, after which data was exported to Microsoft Excel and processed to determine relative abundance levels. Means were compared with unpaired *t*-tests using GraphPad Prism software.
